# Farmers' Decision Making on Livestock Trading Practices: Cowshed Culture and Behavioral Triggers Amongst New Zealand Dairy Farmers

**DOI:** 10.3389/fvets.2019.00320

**Published:** 2019-09-20

**Authors:** Arata Hidano, M. Carolyn Gates, Gareth Enticott

**Affiliations:** ^1^EpiCentre, School of Veterinary Science, Massey University, Palmerston North, New Zealand; ^2^Cardiff School of Geography and Planning, Cardiff University, Cardiff, United Kingdom

**Keywords:** culture, livestock trading, livestock disease, middle-man, stock agent, behavior, behavioral change, qualitative interview

## Abstract

Studies of farmers' failure to implement biosecurity practices frequently frame their behavior as a lack of intention. More recent studies have argued that farmers' behaviors should be conceptualized as emergent from farming experiences rather than a direct consequence of specific intentions. Drawing on the concepts of “cowshed” culture and the “Trigger Change Model,” we explore how farmers' livestock purchasing behavior is shaped by farms' natural and physical environments and identify what triggers behavioral change amongst farmers. Using bovine tuberculosis (bTB) in New Zealand as a case example, qualitative research was conducted with 15 New Zealand dairy producers with varying bTB experiences. We show how farmers' livestock purchasing behavior evolve with culture under a given farm environment. However, established cultures may be disrupted by various triggers such as disease outbreaks, introductions of animals with undesired characteristics, and farm relocation. While dealing with economic and socio-emotional impacts posed by triggers, farmers reorganize their culture and trading behaviors, which may involve holistic biosecurity strategies. Nevertheless, we also show that these triggers instigate only small behavioral changes for some farmers, suggesting the role of the trigger is likely to be context-dependent. Using voluntary disease control schemes such as providing disease status of source farms has attracted great interest as a driver of behavioral change. One hopes such schemes are easily integrated into existing farm practices, however, we speculate such an integration is challenging for many farmers due to path-dependency. We therefore argue that these schemes may fail to bring their intended behavioral changes without a greater understanding of how different types of triggers work in different situations. We need a paradigm shift in how we frame farmers' livestock trading practices. Otherwise, we may not able to answer our questions about farm biosecurity if we continue to approaching these questions solely from a biosecurity point of view.

## Introduction

Theoretical and empirical research studies have shown that farmers' practices play a substantial role in determining how livestock diseases spread within and between farms (1–4). In particular, farmers' livestock trading behavior can be responsible for the geographical spread or translocation of disease (5, 6). Previous studies have suggested that regional or national-level livestock movement patterns are sensitive to externalities such as an imposition of new legislation and changes in global milk prices (4, 7–9). However, despite epidemiologists' use of social network analysis to understand the temporal and spatial variability of movement patterns (10), there is surprisingly little research that seeks to understand how and why individual farmers make livestock trading decisions (11). This paper seeks to address this gap.

Literature on livestock trading practice almost exclusively frames farmer behavior from a biosecurity perspective. Given that livestock trading is one of primary means of introducing a disease onto a farm, it is natural that this framing is popular. Under this framing, various practices associated with livestock trading have been previously studied including maintaining a closed herd (12, 13), verifying the disease status of purchased animals (14–16), and considering the disease risk status of source farms and regions (10, 17). Other studies suggested that farmers perceive these practices as being effective, but often impractical (18), which may partially explain why farmers do not often implement these measures. These studies often use behavioralist approaches that focus on the motives, values, and attitudes that determine farmers' decisions. Quantitative methodologies associated with psychological behavioral theories such as the Theory of Reasoned Action (TORA) or Theory of Planned Behavior (TPB) (19, 20) have been widely used in studies of farmer behavior (21–23) allowing policy makers to hone key messages to farmers in order to change their behavior (24). However, Burton (25) cites a range of conceptual and methodological problems associated with their (mis)use in agricultural behavior studies, including; failure to take into account the influence of significant others by conflating subjective norms with attitudes; failure to take into account specific contexts or the “compatibility principle” when analyzing the influence of others (26, 27), and the time and resources to capture appropriate data (28).

More recent studies have suggested that other factors beyond farmers' attitudes toward biosecurity, contribute to livestock trading behaviors. For example, some studies indicate that farmers' physical and environmental conditions play an important role in shaping their behaviors (29) whilst others demonstrate how social, physical, and biological factors collectively influence farmers' behavior (30). These approaches emphasize how farmers' behavior is not a result of specific intentions, but emerges from deeply embedded, path-dependent and location specific farming cultures, or what Burton et al. (30) call “cowshed” cultures. Sutherland et al. further proposed the “Trigger Change Model” to explain a mechanism by which a major change occurs in such culturally-embedded farm practices (31).

Using data from qualitative interviews with 15 dairy producers in New Zealand, and drawing on the concept of cowshed culture, this paper first shows how farmers' livestock trading practices are developed and maintained. Then, drawing on the Trigger Change Model, we further explore how these behaviors are disrupted and reorganized in relation to the management of diseases, particularly bovine tuberculosis (bTB). The paper begins by providing further details on the conceptual framework, before detailing the methodology and discussing the results.

## Methodology

### Conceptual Framework

#### Path-Dependency and Cowshed Culture

The development of cultural approaches to understanding farmer behavior has been a reaction to behavioralist approaches (32). For Burton (24, p. 365), various challenges associated with these approaches lead to a failure to produce data “capable of producing a broad enough picture of farmer motivation.” Instead, he argues for an approach that incorporates the importance of the “self-concept” and “self-identity” (33). Burton argues that farming is “heavily imbued with status symbols” which contribute to the notion of “good farming” and the “good farmer” which play an important role in guiding and shaping farmer behavior (34–37). Status in agriculture is linked to the practical skills and abilities that constitute a “good farmer.” Frequently, these abilities are linked to the ability to maintain “tidy landscapes,” produce quality livestock or operate a successful business objectified through new machinery (37–39). The open nature of farming allows farmers to constantly examine other farms for the symbols of good farming—a process known as “hedgerow farming”—such as maintaining tidy farm yards, planting crops in straight lines, and/or maintaining effective stock fences (36). The absence of this symbolic capital leads to low status and damages the reputation of the farmer. The failure of agri-environment schemes to develop broader cultural change may therefore reflect a lack of recognition of the importance of these cultural symbols (38, 40). Similarly, a recent study showed that the concept of self-identity is also important in explaining farmers' biosecurity practices such as reporting and prevention of exotic diseases (41).

**Figure 1:**
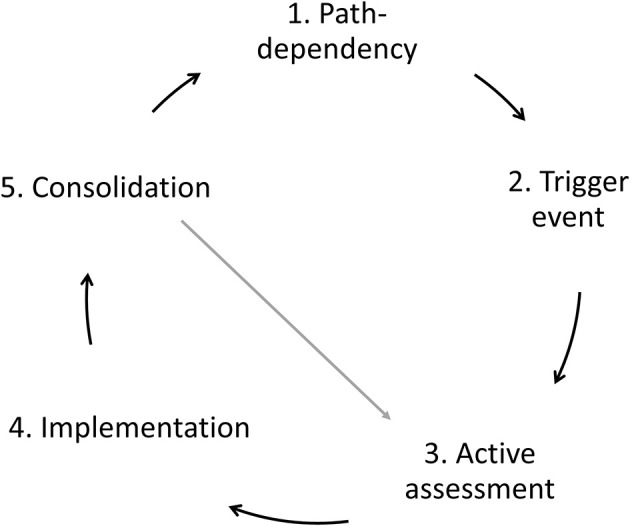
The “Triggering change” model redrawn from Sutherland et al. (31).

Models of farming change and transition also emphasize the significance of self-identity. For example, Sutherland et al.'s (31) model of farming change (see Figure 1) begins with the premise that farmer behavior is path-dependent and locked into social, material, natural, and economic relationships that guide and legitimize existing farm practices. These “socio-technical lock-ins” are difficult to escape: farmers are locked into markets and required to meet contractual arrangements for which they have invested in technological systems. This kind of technological lock-in may also be accompanied by knowledge path-dependency. Here farmers develop forms of practical “know-how” (42) taking routine advice from trusted knowledge sources but which may limit their ability to respond to new challenges (31). Therefore, path-dependency can be expressed in various forms. It may exhibit as a behavioral form, where farmers are locked into specific farm management practices. Or, it can take a social form—farmers may be locked-in specific beliefs or morals.

Path-dependency and the significance of cultures of good farming should not however be seen as simply a social construction. Drawing on recent post-human analyses of farming conduct (43), Burton et al. (30) incorporate the non-human into farming cultures. In this view, farm animals and farming materialities (farm sheds, milking equipment, ear tags, and fields) contribute to the relational construction of farming culture. Segerdahl (44), Hemsworth and Coleman (45), and Burton et al. [(30), p. 176] argue that these relations construct “a human/animal culture with each farm developing its own particular culture as a result of interactions between humans, livestock and the farm buildings.” These relationships are constantly in the making and are influenced by neighboring farm cultures, but collectively form what Burton et al. refer to as “cowshed” cultures which provide each farm with its own distinct path or trajectory (30).

Drawing on these perspectives, we frame farmers' behaviors as shaped and locked-in by various factors including their self-identity, belief, farm environments, and farmer-animal relationships, which are referred to as cowshed culture.

#### The Trigger Change Model

One challenge facing cultural theories of farmer behavior is working out under what circumstances farmer behavior changes. According to the “Trigger Change Model” (31), path-dependencies may be challenged by “trigger events” which create windows of opportunity for farmers to change practices. Triggers may be positive or negative, singular or multiple and may accumulate over time or represent a shock event. Sutherland et al. identified three broad categories of triggers. First, triggers relating to the farm business such as commodity prices, land availability, or regulations. Second, those relating to the life course of the farm household such as retirement, unexpected injury or death, and fluctuations in labor availability. Finally, triggers may relate to challenges to farmers' moral beliefs about the purpose and practice of farming which may arise following disease outbreaks (46). Triggers prompt an assessment of options but Sutherland et al. stress that this is not linear, and may occur over several years during which a passive approach to problems alternates with active appraisal of options (31). For some farmers, assessment of options may involve active experimentation, whilst others seek professional advice, or speak to other farmers. Change may therefore be an incremental process rather than a radical switching between different options and farmers may return to actively assessing practices to assist the consolidation process. Drawing on the Trigger Change Model, we explored triggers that disrupt the path-dependency phase and instigate changes in farmers' livestock trading practices.

### Study Context

#### Institutional Structure of New Zealand Dairy Farming

Two distinct features of the New Zealand dairy farming system make it suitable to study stockpersons' livestock trading decision-making. First, almost all New Zealand dairy farms run an extensive seasonal pastured-based system, where farmers heavily rely on the growth of pasture for animal nutrition. Second, the majority of milk produced in New Zealand is exported to an international market, meaning that the financial status of dairy farms is substantially influenced by international milk prices. These two uncontrollable external factors (weather and international market price) are dynamic and to some extent unpredictable. New Zealand dairy farms therefore need to manage their systems flexibly according to the changing situation. In particular, farmers are required to continuously adjust their herd sizes: the size often needs to go down if there is insufficient pasture to minimize a running cost and go up when a milk price is higher to increase a profit. This leads to dynamic and frequent livestock movements throughout the country. The need for a dynamic change in a herd size also provides difficulties for dairy producers because their trading events are irregular in terms of size and timing. For instance in UK, stockpersons may be able to trade with the same partners over years (17). In such a situation, studying farmers' decision making may not be straightforward because trading livestock with an established partner can be merely a routine such that farmers do not have to consider, if any, factors in relation to trading. On the other hand, New Zealand dairy producers may have to identify a new partner at every trading event (this need is repeatedly mentioned in our interviews shown below). Taken together, the New Zealand dairy farming system therefore offers a distinct opportunity to understand the development process of livestock trading decision-making. This does not, however, preclude applicability of our findings to other countries (see Discussion).

#### Bovine Tuberculosis in New Zealand

Bovine tuberculosis (bTB) in livestock is designated a notifiable disease in New Zealand. Herds identified with bTB are required to immediately cull bTB positive animals and are placed under cattle movement restrictions until the disease is cleared, which can cause significant economic burdens for affected farms. New Zealand has succeeded in substantially reducing the number of bTB infected livestock herds based on various control strategies (47). Regionalization and risk-based trading schemes are assumed to have played a pivotal role in preventing a bTB spread between herds (10, 29, 48). In this context, regionalization categorizes livestock herds into several groups primarily based on the risk of bTB infection in their geographical area. Previous research has found evidence that this may result in risk-averse purchasing practice where farmers in low risk regions avoid purchasing cattle from high risk regions (10). In contrast, the risk-based bTB trading scheme in New Zealand reveals whether or not a farm is currently infected with bTB, and confers a number (maximum 10) to each bTB free farm to indicate how many years the farm has been bTB-free. This system, referred to as C status, may provide stockpersons with further information regarding a bTB risk; however, in areas of historic high bTB prevalence, stockpersons' experiences of disease incidents mediates the meaning and understanding of C status, affecting their herd management decisions (29). Regionalization and C status therefore provide an opportunity to analyse how disease risk information affects farmers' livestock purchasing practices.

### Qualitative Interviews With Farmers

Data were collected from 15 qualitative interviews with New Zealand dairy producers. New Zealand dairy producers can be categorized into three groups: farm operator, share-milker, and worker. A farm operator owns both the cattle and the land and may hire additional workers. A share-milker owns the cattle, but not the land, and therefore leases infrastructure (e.g., land and cowsheds). A common type of share-milker is a so-called fifty-fifty share-milker, who receives 50% of the total profit from the milk production. A worker includes those who work for either farm operators or share-milkers and do not own either the cattle or the land. In this study, we included both farm operators and share-milkers since they are responsible for making decisions around livestock trade—hereafter, we refer them to as farmer.

**Figure 2:**
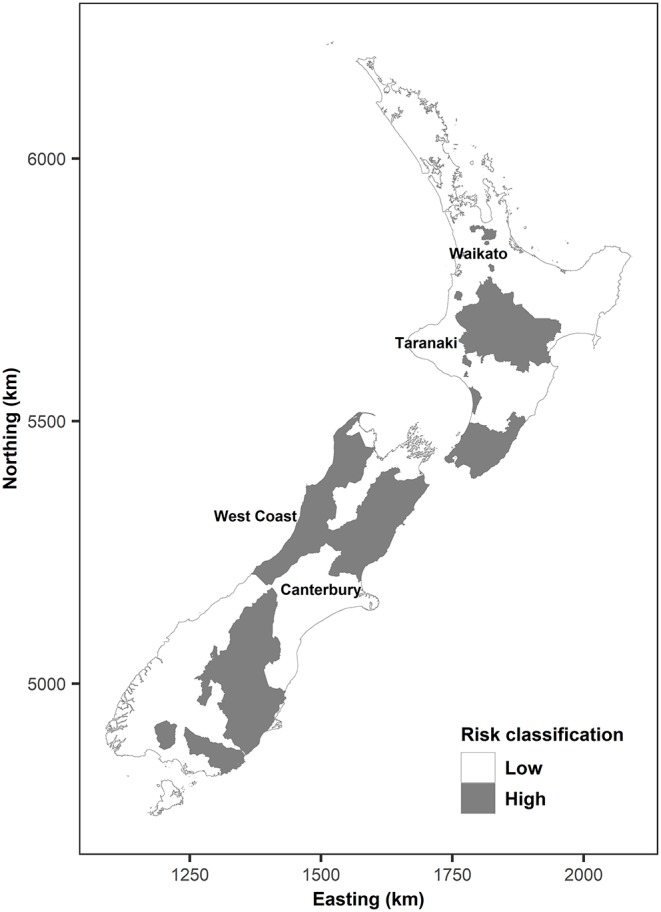
Locations of regions from which interviewed farms were selected in relation to bTB risk. Note the current high bTB risk area as of 2019 is smaller than shown in this map.

The interviewed farmers included individuals from both low and high bTB risk areas to investigate differences in how they develop a livestock purchasing strategy. For a low bTB risk area, we purposively chose Waikato, Taranaki (North Island), and Canterbury (South Island) because these are the major dairy producing areas in New Zealand (49). For a high bTB risk area, we chose West Coast (South Island), which has maintained one of the highest prevalence of bTB in New Zealand over several decades (47, 50). Figure 2 depicts each region in relation to bTB risk. Our sample size of 15 was determined to maximize the sample size within the budget and time. We aimed to obtain the size larger than 12 based on findings from Guest et al. (51) that data saturation in qualitative interviews can occur at the sample size of 12; this was also shown in other recent studies of farmers' decision making and disease control (18). The sampling frame was generated by asking researchers, veterinarians, and industry stakeholders to provide a list of candidate stockpersons in each region that may be willing to participate in the study. We also contacted individuals in OSPRI—the organization responsible for bTB control in New Zealand—to provide a list of farmers who had previously experienced a bTB breakdown and would be willing to participate in this study.

All potential participants were contacted by phone and the objective of the study (i.e., livestock trading decision making) was explained. After their willingness to participate was confirmed, in-depth face-to-face interviews were carried out between November and December 2016 at the interviewee's preferred location which in all but one case was the farm property. Interviews lasted between 30 and 83 min. Two interviews were conducted with female farmers, 12 interviews were conducted with male farmers, and one conducted with a husband and wife couple. The profile of interviewed farmers is summarized in Table 1. The interviews were semi-structured whereby farmers were initially asked several questions about background information of themselves and their farms. Interviewees were then asked if they had purchased or sold any cattle recently and if so they were asked to tell stories about the experience. Subsequently, depending on how interviewees responded, different lines of enquiry were used to ask the following questions; how and when they made a purchasing and/or selling decision; any experience that changed their trading practices. All interviews were conducted by the first author. To compensate interviewees for their time, a NZ$100 gift card was given to each participant after the interview.

**Table 1:** Profile of interviewed farmers.

**Farmer**	**Region**	**Type**	**Number of milking cows**
1	Canterbury	Farm owner/operator	1,500
2	Waikato	Share-milker	420
3	Waikato	Share-milker	330
4	West Coast	Farm owner/operator	175
5	Taranaki	Farm owner/operator	440
6	Canterbury	Farm owner/operator	2,400
7	Waikato	Farm owner/operator	624
8	Canterbury	Farm owner/operator	2,700
9	Canterbury	Farm owner/operator	1,500
10	Taranaki	Farm owner/operator	350
11	Waikato	Farm owner/operator	3,500
12	Canterbury	Share-milker	900
13	West Coast	Farm owner/operator	184
14	West Coast	Farm owner/operator	580
15	West Coast	Farm owner/operator	440

### Analysis

Interviews were all audio-recorded and transcribed by the first author. Personal identifiers were removed from the transcribed files to ensure the anonymity of interviewees. Transcripts were imported into NVivo Pro 11 for Windows (QRS International, Australia). Data was analyzed using thematic analysis drawing on the concept of cowshed culture and the Trigger Change Model as described above. The transcripts were coded and then clustered into themes, whose inter-relationship was subsequently analyzed.

## Results

Analysis of interviews focused on how farmers' livestock trading behaviors are shaped by the four key stages of a farm culture development—emergence of cowshed culture, path-dependency period, trigger events that disrupt existing cowshed culture, and recovery from the disruption. The following details how each of these stages influence farmers' livestock purchasing practices.

### Shaping Cowshed Culture: Contributions of Physical and Natural Farm Environment

Although “hard work” is a characteristic of farming cultures (34), the theme of “making things easy” was frequently mentioned by farmers in interviews. Specifically, “making things easy” referred to two key components in farm management: firstly, developing and maintaining a smooth milking flow. This referred to the ability to milk cows as quickly and efficiently as possible. Secondly, developing and maintaining smooth pasture grazing management. This referred to the ability to flexibly manage the grazing intensity and area on pasture to maximize its quality while meeting the energy requirement of cows to secure sufficient milk production. Farmers therefore try to develop farming practices that enable these two components, creating a cowshed culture specific to each farm. Our analysis highlighted that both physical and natural farm environments play a role in shaping farmers' management practices.

#### Developing a Smooth Milking Flow

The following extract of farmer 1 (Canterbury) exemplifies the importance of a physical environment in shaping farmers' behaviors.

F: “When we take the heifers into the herd for milking in their first lactation, we will split them between 2 sheds on breed. Because this shed down here is rotary with grain feeding, short tracks… so the tracks aren't very long and very good tracks. So we put the all Friesian, the big cows, down here. And the other shed, it's a herringbone shed, old cowshed. Not made for big cows with no grain feeding. Very long walks and the tracks aren't quite as good. So we put the cross-bred and Jersey, anything with harder feet, we put them in this shed […].”

I: “So they rarely mix?”

F: “No. […] It just makes the management easier when you have all your cows are the same. All these cows are roughly the same size, uhmm, and all cross-breds, all black and brown, and when they line up in the herringbone it's easier to have whole lot of cows the same than just to have big cows and small cows and… or whole big cows and try to fit little one in the middle… they don't like it. If you keep them all the same, it's nicer for them, they fit better.”

Interactions between the material farming infrastructure (cowsheds and walking tracks) and the behavior of cattle, in turn shapes farmers' herd management decisions. In doing so, cattle are less likely to have lameness and feel less stress during milking, contributing a smoother milking flow which saves farmers time and stress.

#### Developing a Smooth Pasture Grazing Flow

Many New Zealand dairy producers run an extensive pasture-based grazing system. The seasonal weather patterns distinct to each region affect the growth of grass and paddock conditions. Grazing is not only about feeding cattle in New Zealand; but it is an important part of farming to control the quality and growth of grass (52). Grazing with too much intensity may damage the soil and grass, and poor paddock conditions may lead to lameness in cattle, which disrupts milking flow. A successful understanding of this complex relationship enables stockpersons to manage a farm better. For instance, farmer 4 in West Coast, which has high rainfall, explained how their cattle stocking rate is determined by the weather:

“That would be a typical rate around here, about 2 to 2.3 [cows per ha] maybe. Because you know when it gets, I mean if you get a year what you would consider to be drier, then everything is going good… you would think oh you know we could run probably 3 cows to ha and probably you could. But then it'll go bad and you wish you had known. One of the neighbors up road said to me “Oh we run about 2.1”. And I thought “It's not many”. But after being here for 7 years I can see why. You don't have too many cows over here. Because when it gets wet there is nowhere to put them.”

Importantly, these cowshed cultures emerge over time and may take many years to develop and become established. A cowshed culture specific to each farm contributes to various farming practices such as which cattle breed and how many of them to keep and how to manage them, which in turn guides farmers' livestock trading practices. For instance, farmer 11 (Waikato) explained how his observations of cattle behaviors in his natural farming environment shaped his decision to purchase from farms that have similarly hilly paddocks in Palmerston North−300 km apart from his farm—rather than Morrinsville, which is one of major dairying areas nearest to his farm.

### Path-Dependency

Our analysis highlighted that a specific path-dependency is created through interactions between various factors including physical and natural farm environments and farmers' beliefs. Firstly, decisions to purchase cattle are guided by the cowshed culture of each farm. For instance, share-milker 12 demonstrated how his choice of livestock to purchase is dependent upon the interactions between cows and the material design of his milking shed:

“[…] you've got things like [which] cowshed they are coming from as well… like herringbone or rotary… there are always things you got to think about. Some sheds go clockwise and somewhere anti-clockwise. […] You are still gonna disrupt the cow flow when you are training them, yeah it makes a difference. Just a little thing that people are not always interested in. Practical things, you can't explain all these things.”

This extract further emphasizes that this farmer's behavior is guided by his practical capital: skills that are “difficult to explain” but understood by farmers. Such practical capital, or “know-how,” may arise through experiencing “what works and does not work” under their material and natural farming conditions (31). The ecology of each farm also contributes to creating a path-dependency. For instance, farmer 10 explained how the availability of fodder in the pasture he owns determined his farming practices, creating path-dependent livestock trading behaviors:

“We have to really [buy replacement animals] because as I say we are selling out cows every year, we haven't got enough cows to supply all our extra replacement that's why… if we weren't selling the cows, we are good to be our own. But we are selling cows we have to buy… especially the grazing block, to keep that fully functioning, we need so many stock. If we had our own herd and we don't sell anything out every year we kept them all and certainly we could have our own… numbers and replacement so we could be selling extra heifers each year but…”.

This farmer indicated in the interview that he had been selling almost half of his milking cows every season in the past several years because there had been a continuous demand of a large number of cows from South Island farmers. This selling practice, however, results in a shortage of replacements because not all of remaining cows are artificially inseminated hence their calves may not be suitable as replacements (calves from cows that are not artificially inseminated usually have inferior genetic merits and lower milk production). However, the extra paddock he owns allows him to purchase a large number of calves and heifers, which will serve as replacements. This system was proven to be profitable, therefore, he is “locked-in” in the situation where he continues to purchase and sell livestock, although he theoretically has an option to have a closed herd. Path-dependency is therefore not necessarily inefficient: some farmers believe that being on a path-dependent farming trajectory is important. For example, farmer 9 explained he is trying to achieve the maximum potential of his herd by breeding only animals which perform well in his specific farm environment and management practices, instead of introducing animals with better genetic merits.

### Triggers and Disruptions to Farming Cultures

Interviews revealed several triggers that disrupt cowshed cultures and alter livestock purchasing practices. Firstly, relocation to another farm was a significant factor in triggering reassessment of existing practices. The role of share-milking in the New Zealand dairy industry means that relocating a herd can be a common practice, with herd relocation occurring annually on June 1st—referred to as “gypsy day”—when existing share-milking contracts end and new ones begin. Given the significance that farm environments play an important role in shaping cowshed culture and farm practice, ending a share-milking contract may provide an opportunity to develop new farming practices. However, moving may also trigger further complications where the fit between new and old cowshed cultures is poor. For example, as a share-milker, farmer 3 needed to relocate to a new farm and he noted that they were trying to down-scale the size of animals in his herd after the relocation:

“Main reason we wanna bring the size of the animals down is… cos the cows are getting too big and this farm gets quite wet in winter and big cows are gonna sink, so they get a lot of lame feet, and…. Little cows just seem to be more profitable… it is lighter on feet and easy to maintain.”

In this example, new farm environments provide opportunities to see how the relationship with existing cows results in new challenges, and the need to change the kind of animals reared.

Secondly, the share-milker system may also act as a trigger to land owners themselves who contract share-milkers. While share-milkers' goals are often to produce sufficient amount of milk in each season so that they can save money to buy their own land in future, land-owners may have a longer-term priority such as maintaining pasture quality:

“yeah [I own] all the cows, the farm owner owns the land. They live in the next farm. Some farm decisions we make together…cow number… we make budgets. There's lot of communications there. We have to do a weekly report. Like emailing every the other day. Because they don't own the cows… they like to know all these information…. But you've got to communicate… it's hard cos they're running other business… They come and see farms in a different angle cos they don't know all the practical things…. Running the cowshed and managing the staff…they never milked before. (Share-milker 12)”

This extract highlights this share-milker's frustrations and difficulties in communicating with land-owners who “do not know practical things”— the difficulty in creating and maintaining material (running cowshed) and social (managing staff) aspects of cowshed culture. But this extract also clearly highlights that the difference in their background and business goals also create frustrations in land-owners. These frustrations may accumulate over time, and can act as a trigger event either by looking for a new share-milking partner, or by taking control of the farm management completely. For example:

“…until 7 years ago we didn't own cows… any dairy animals at all. We had a 50-50 share milker on here so they owned all the livestock and then we've done that for 12 years… decided we want to more control… and we're going to put a management on… but obviously that meant we had to buy cows, buy more machineries, need to hire staff… so went on and bought a whole herd of cows in one year for that farm… and then we went to do the same thing following year for the new conversion. So we bought 1200 and something cows and it took 2 years to get these 2 farms up running… so it kind of went from not being a dairy but having a dairy to put all in (Farmer 1).”

Thirdly, the arrival of new cattle onto a farm—either due to the relocation of a new share-milker or the routine purchase of replacement cattle—can lead to triggering events. Purchasing livestock can disrupt an established farm management flow for various reasons, and this can repeatedly pose physical and psychological stress on farmers, which act as a trigger. For instance, share-milker 2 demonstrated how a disruption in the milking flow due to introduced cattle stressed him, which made him reluctant to purchase livestock anymore:

“Because our shed's quite unusual, you don't get too many internal rotaries. […] there's not many sheds like this so there's not many cows that know how to come…that's another thing that stops me from trading is that it's bloody hard to teach cows to come in the shed. So you can train them how to do that… so it took us 3 months to teach them how to come in. And even then after years some cows don't wanna come in.”

Introducing external cattle can also bring diseases onto a farm, which can cause a substantial disruption to cowshed culture. For example, a bTB breakdown leads to livestock culling, if not a whole herd, and restrictions on selling and moving animals. The latter can be particularly critical for New Zealand dairy producers because selling and moving animals to other properties is an important herd management practice when the fodder is limited. Farmer 5 demonstrated how the bTB breakdown imposed not only an economic, but also a psychological distress by limiting his farming options:

“When you've got no option, you got into a corner… it's kind of sucks. When you've got option, you're always on the front foot, thinking about what you can do next, and that's kind of where we've got to in the last 12 months. And the part of that is changing the whole farm system. So you know… last 2, 3 years I felt like a death by thousand cuts type things… slow way of dying… you're always fighting fires… you're always wondering where how your next dollars are coming from… whereas if you've got options in your back pocket, then all of sudden your attitude can change. From fighting fires to actually thinking ‘Ok where the hell am I going now? What am I gonna do?' And it's easy to say just a mindset but it's actually more than that. To get that mindset you need the options to start with. You can say ‘Well…get the mindset and options will come' but it doesn't always work out. You know sometimes mindset is because of lack of options.”

This extract demonstrates how the farmer struggled to be economically viable after the bTB breakdown due to various restrictions. The farmer described that he had been in “thinking in a silo mentality,” where he tried an incremental small change to his farming practices but they did not improve the situation. This imposed a psychological distress and the accumulation of these experiences acted as a trigger. The farmer finally succeeded to turn over this situation by changing the whole farming system.

### Response to Triggers: Active Assessment of Alternatives and Implementation

In response to trigger events, farmers may start assessing options more actively. Sutherland et al. argue that farmers are more motivated in this period to consider a wide range of alternative options and information compared to when they are at the path-dependency stage. As a result, farmers may change their practices or beliefs but the approaches farmers take may vary considerably (31). As summarized in Table 2, we identified several farmers' responses to specific trigger events. However, in general, interview data showed two clear long-term strategies for responding to triggers associated with the movement of animals: firstly, the use and mediation of cattle disease risk scores; and secondly, the use of stock agents. Both strategies demonstrate how farmers' decision-making evolves and consolidates over time in relation to other social, natural and material dimensions of cowshed culture. Moreover, each strategy seeks to maintain or restore an equilibrium to cowshed culture through purchasing practices. Details on each strategy are found below.

**Table 2:** Examples of trigger events and accompanying responses made by farmers.

**Examples of trigger event**	**Example of the response and quotes**
Livestock introduction	Stop purchasing specific animals
	“*…we had bulls last year that had a bloody pink eye. Bad… bad strain of pink eye [infectious bovine keratoconjunctivitis]. So we had some teaser bulls [for a heat detection] last year. So decided not to use teaser bull ever again for that reason because…[…]. I mean the benefit of them is not worth for the risk. So we got about 60 – 70 cows with pink eye in the herd the other side of the road last year and we were very careful not to let any of these cows from this farm mingle with those ones to cross infect. Uhmm I think we've got under control now, but uhmm… it was you know the guys had to be very vigilant looking at eyes and making sure that they treated them. […] It was more just … hassle and cost… and stress because you know that they could go through the whole herd and imagine you'd have to put stuff on eyes on every cow… nah.”* (farmer 9)
Livestock selling	Assess the need of a disease control after having been frequently requested by buyers for the disease status of animals the farmer was selling
	“*It's something I've never worried too much about, but it's something that are starting to look at more… Because I just had one reactor, get rid of it yesterday. It's probably something we would check… I know it's becoming more…. When we sold cows last year, they wanted BVD status, the history, the records, so yes. [….] I think many years ago I've got herds of heifers out for grazing and quite a few was empty… 8 or 10 empty heifers and we reckon that was BVD that has been spread…”* (farmer 10)
Disease outbreak	Purchase new pasture (a run-off) that allows a farmer to stabilize the farm business
	“*No [I'm not allowed to sell animals] and I'm not allowed to put animals for grazing. But like I say, that's not a problem. I can live with that in a management issue. And that's what I'm saying, thinking farmers that get TB… I highly recommend they become independent. Not really nice but you really do have to operate your farm inside the silo. And that [having their own run-off] means you're not paying grazing anymore. You've got to pay interest on a grass, better to make that decision.”* (farmer 5)

#### Using and Translating Official Disease Information

In response to the impact of cattle movements and disease outbreaks, farmers seek to adapt their cattle purchasing decisions through a process of actively assessing their own experiences of disease with official information. Interviews with farmers clearly highlighted the impact of trigger events on bTB risk management, as summarized in Table 3. Farmers in low bTB risk regions and without experience of a bTB breakdown may not actively assess the importance of C status as long as a source farm is free from bTB. Nevertheless, farmers seem to change the interpretation of the C status after trigger events including a bTB breakdown and farm relocation from a low to high bTB risk region; the C status is no longer just a number but information that need to be interpreted for each farm.

**Table 3:** A summary of quotes on the C status from farmers stratified by the risk of bTB in their farming regions and the presence of a bTB breakdown experience.

	**No bTB breakdown experience**	**bTB breakdown experiences**
Low bTB risk region	“*As long as they're passing TB test… yeah as long as they pass TB test I don't think I'm too worried. I've never really thought about it. As long as they're clear and not on movement control… that's not a factor when I buy animals… definitely I don't wanna get TB”* (share-milker 2) “*…as long as they're clear yeah, it's all good. I haven't looked at it too closely. Because most of us are [C]10 here.”* (share-milker 3)	“*Probably didn't worry about that back then [before the bTB breakdown], didn't really think too much about it [source farm C status]. I just presumed if they were clear, they were clear you know. But probably just now look at the history and where they come from and …, how long they have been on that farm and where they are buying from… share-milkers move around obviously quite a lot so you have to be careful about that.”* (farmer 6)
High bTB risk region	“*…we bought C1 [a herd that just became clear for bTB a year ago] at the first year we were here [after having moved from Canterbury, which is a low bTB risk area]. And sort of I wished ever since we hadn't but anyway we didn't get TB, touch wood, as far as we know. We haven't had any since we've been here. Yeah I wouldn't do that again. I wouldn't buy C1 again, ever. It's just too risky.”* (farmer 4)	“*Depends where they are and why they are [with a specific C status]. You know, you look into those sorts of things. And where they are coming from… like here in the coast, it's a TB area so you know that it would be the likelihood but… yeah we just go through… check it out.”* (farmer 14)

#### Shady Farmers and Trusted Stock Agents

The second strategy farmers employ is developing a trusting relationship with stock agents who can help farmers source replacement cattle to fit their cowshed cultures. As we describe below, this strategy helps farmers to avoid purchasing from “shady” farms, which was revealed to be a common concern for farmers. Farmers often demonstrated that unless they are exiting the dairy industry, they normally send cattle that are unproductive or have serious health conditions (i.e., repeated mastitis and lameness, and behavioral issues) to slaughter and sell cattle that can still produce milk but only at a suboptimal level on their farms. Nevertheless, they also often noted their concerns about the presence of other farmers that sell cattle which should have been sent to slaughter. This is problematic for farmers; it is difficult to notice these serious mal-conditions when purchasing because it takes a while to recognize these problems or requires an observation under a specific circumstance such as during milking, as illustrated by following extracts.

“Three quarters […] people don't want those. Off to the works. Mastitis definitely. We would not knowingly sell cows that has got mastitis or repeated lameness, we wouldn't do that. That's not honest. That's a very shady farmer that would buy those and if he is shady he's got selling to somebody else. And our industry needs that… we need to be self-monitoring. We need to be able to trust each other. We don't need shady people. Cos it's a very hard industry to be in.” (Farmer 11)

“I don't actually like sale yards […] you don't really know why those animals are on sale yards sometimes. Fine you might look at these animals and the animals are perfectly healthy. These animals might have been sent to the sale yard to go to the works because they've got problems.” (Farmer 5)

Farmers seem to have various approaches to avoiding shady farmers including personal trading and using stock agents, as summarized in Table 4. While the use of a stock agent seems to be common among New Zealand dairy farmers, the extent to which farmers rely on stock agents in deciding which animals to purchase varies. While some farmers mentioned they do not even see animals which agents chose for them before purchasing, some farmers make sure they visit and check the selling farm—this is a further strategy to assess whether the seller is honest and has a good cowshed culture. This assessment involves either communicating with the seller or visually checking the farm and cattle, or both. For example, share-milker 3 noted:

“He [stock agent] sort of got…3 or 4 herds for me to look at and we went for a drive one day. I think we went to…the first 3 and I was like ‘Hmm, I hope the last one is good'. […] The way the farmer had them… it wasn't… they were a little bit light and looked ugly. And rough… the coats were rough. They weren't shiny, healthy looking. So it just sort of gives you an idea that maybe he doesn't do job properly. When we went to the last one the owner came with us we went around and he told me this cow doesn't give much production, this is my peak cow here. You know he just knew his herd. He looked like he had more involvement with it and he actually cared. As soon as I walked in there I was like this is what I want. It's a nice looking herd.”

This quote highlights two important points. First, the farmer assessed the sellers' farm management as poor based on the “ugly” appearance of their cattle, reflecting the role of “hedgerow farming” and appearance of livestock as ways of telling apart “good” farmers (18, 36). The “ugly” appearance of livestock therefore indicates farmers' poor management and hence links to “shady” farm culture—cattle on these farms may have some hidden problems. The link between the poor animal care, poor management, and “shady” farm culture is also mentioned by farmer 14: “if he is not looking after his animals and records probably are not 100% either.” Second, “knowing their own herd” provided the farmer with a credential that the seller is genuine. Farmers who know their own herds well are likely to be able to identify problems in cattle quickly and minimize stress on cattle, which is an important component of a good farm culture (30).

**Table 4:** Advantage and disadvantage of identified methods to avoid shady farmers and associated farmers' quotes.

	**Using stock agents**	**Personal trading**
Advantage	1. Stock agents in general have good knowledge about sellers locally and nationally.	The sellers can be trustable if farmers know the seller personally.
	“*he [stock agent] knows… ‘this guy is selling cows, selling surplus cows for 5 years or 10 years and we never had problems or he sold some cows and we had a bit of problem 3 years ago so maybe you don't wanna go there'… so he knows all that. Whereas if we're going to trying to deal with other farmer, they don't tell you, you won't know.”* (farmer 4) “*Yeah, at the moment we are looking for 50 more cows. Because we need to keep the numbers for the contract for the farm owners. But there's no. not much stock in Canterbury…so we're looking in North Island, I think he's [stock agent] in Taranaki now… That's what's going on there. They're busy people, buying around the country looking at animals, but it's good, it's what they do, you know, they're professionals.”* (share-milker 12)	“*I mean we've got neighbors around the road but he's got Friesian. If we wanted to buy Friesian, I'm happy to buy them off from him. Because he thinks the same as we do. […] Honesty, integrity, you know, if there was a problem he would tell you what it was.”* (farmer 4)
	2. Stock agents solve issues around trading between farmers, including a price negotiation.	
	“*We… a few years ago… we sent some young stock away grazing…grazing that was organized through an agent… the grazing didn't go very well… and we went over there and decided we were taking animals home. […] they were not gaining enough weight fast enough for the money we were paying. […] So our agent… they sorted it out. It was very interesting… dealing with that. I think if that was a private deal without the agent there, without a contract, you would almost don't have legs to stand on.”* (farmer 1)	
Disadvantage	Building a trustworthy relationship with agents may be slow and requires a constant assessment.	It is often infeasible because 1 farmers do not know many sellers who are selling at the right timing (farmer 4, farmer 9) 2 difficult to agree on a price (farmer 8) 3 there is no time to set up a personal deal (share-milker 3, farmer 11)
	“*…I contacted one agent that I only met a couple of times and I said “Do you have any profiles for any heifer calves for sale?” and I said I like high index Jersey and he sent me through a profile and they were really average. […] But now he knows that… if I ask him again he would tell me… only give me a higher one because he knows now that his missed out one because I didn't buy them in the end […] When you get to know them, they know you and your farming system as well.”* (farmer 7)	

In summary, purchasing cattle from a genuine cowshed culture is important: animals from such a farm are less likely to have serious problems. Farmers consider good-looking animals, other farmers' knowledge on their own herds, and farmers that care for their animals to be indicative of a genuine cowshed culture. Farmers have various strategies to find such source farms including using a stock-agent, which helps farmers to keep a consistent farm trajectory and new path dependency.

## Discussion

In this section, we discuss how our findings inform understanding of farmers' livestock purchasing behaviors.

### Trigger Change Model

Three important points can be drawn from our findings in relation to using the Trigger Change Model to assess farmers' behavior related to disease management. First, our research confirms that farmers' livestock purchasing practices can become locked in and difficult for farmers to change for reasons such as specific farm material infrastructures (cowshed and walking tracks), natural environment (paddock and weather), and established farmer-livestock relationships. Moreover, farmers may develop favorable beliefs about their practices through repeatedly implementing the practice. Therefore, an apparent lack of adoption of biosecurity practices in livestock trading should not be interpreted simply as a lack of attitudes toward disease control, but rather a reflection of the socio-technical conditions which farmers work within.

Second, voluntary disease control schemes such as farmers revealing the disease status of their farm may fail to induce their intended changes in farmers' behaviors without a greater understanding of trigger events. We demonstrated while some trigger events indeed resulted in a major change in farmers' behaviors, similar events only induced a minor change in other circumstances. This suggests that the impact of triggers is context-dependent. For example, farmers' behavioral response to disease-related events or information likely depends not only on the disease characteristics, but also on a wider range of factors associated with farm circumstance and culture, and livestock trading systems. Together, these reinforce the need to study farm biosecurity practices from a multidisciplinary perspective that includes animal welfare, animal production, and social science rather than solely from a biosecurity point of view.

Thirdly, the model assumes the consolidation phase follows assessment and implementation phases. Our data suggests this separation is hard to detect. Change appears to be an incremental and iterative process rather than a clean break between different options and farmers may return to actively assessing practices to assist the consolidation process. These observations may be partially because we focused on bTB; farmers evaluate the effectiveness of new practice during the consolidation phase, however, the chronic and uncertain nature of bTB, combined with regulations that prevent cattle movements, renders a complete evaluation of whether a new practice is successfully preventing bTB recurrence. In this way, farmers may constantly shift between assessment, implementation and consolidation but without any clear delineation between these phases. Further research is required to establish whether the failure to disentangle these stages of the model applies to other livestock diseases, under which circumstances it is possible to distinguish each phase, and how long each phase may be expected to last.

### How Do Farmers Decide What Kind of Animals to Purchase?

Dairy farming is considered one of the most physically and psychologically challenging jobs (53). The importance of establishing a farm system that enables a smooth, or easier, farm management was often mentioned by the interviewed farmers. Burton et al. argued that an easier farm management leads to happier farm workers and better treatment of cows, which ultimately results in an improved production (30). Indeed, our data showed how farmers try to develop such an easier management system through observing cattle behaviors under their farming environments. This in turn primarily determines the kind of cows to keep on a farm and which cows to purchase. Therefore, livestock purchasing practices seem to be shaped in the process of establishing cowshed culture, rather than farmers choosing “best” cows for their farms after considering a whole range of animal characteristics. This means animal disease status may be dismissed when purchasing animals, although we showed farmers develop various strategies to avoid introducing a disease onto a farm as we discuss later.

### How Do Farmers Know Potential Source Farms to Purchase Animals From?

Our analysis suggested that the use of stock agent in purchasing livestock is common among New Zealand dairy farmers and we argue that this may be one form of the path-dependency. Stock agents come to know what kind of animals farmers are looking for; quality and price of animals, and the fit to each farm's material and natural environment. In turn, this saves farmers' time and, perhaps more importantly, cognitive costs required for decision making. This system is particularly useful for New Zealand dairy farmers because they need to purchase and sell animals flexibly in response to the fluctuations in milk price and weather conditions.

Stock agents work locally and try to match buyers and sellers within a limited geographical area, meaning that trades often occur locally. Occasionally, agents try to purchase animals from other regions when, for example, there are few eligible animals with specific criteria required by buyers. This facilitates a long-distance livestock movement. This indicates that purchasing farmers are often provided options only to purchase animals locally, which may be often beneficial for farmers for two reasons. First, local trading costs purchasing farmers less animal transport fees. Second, farmers in specific climate and environmental conditions may prefer purchasing animals locally, which better adapt to their farm environments.

### How Do Farmers Avoid Introducing a Disease?

Our data suggested that farmers may not be concerned about some diseases that they consider would not disrupt their cowshed cultures. Here, a disruption to a cowshed culture can mean different things to different farmers, although a breakdown of a smooth milking flow may be a significant issue for many farmers; for some a production loss can be a disruption, and for some this may damage a farmer's reputation. This variation may be attributable to various factors including disease experiences, cowshed culture, extra time farmers can spare, and whether they are farm owners or share-milkers. Nevertheless, our study identified several strategies farmers develop to avoid diseases they are concerned.

First, farmers use disease risk score information for bTB (C status). As New Zealand farmers are aware of the serious impact caused by a bTB breakdown and the disease risk score on each farm is relatively accessible, it is not surprising that farmers use this information. However, our analysis showed that the way farmers interpret this information varies between farmers depending on their cowshed culture, disease experiences, and geographical locations, which is supported by a previous finding (29). This emphasizes that farmers do not interpret risk scores linearly, contrary to the way scientists and government officials tend to interpret this information. It is important to understand this non-linearity because a failure to acknowledge this complexity can hinder the success of the voluntary disease control approach that has been of significant interest for governments (17, 54).

Second, farmers may take a more blanket approach to avoid unwanted diseases by avoiding purchasing cattle from so-called “shady farmers” and instead use a stock agent. Farmers demonstrated the difficulty of finding problems in cows before purchasing because the disease status information provided by sellers may be unreliable or diseases associated with milking may only appear in the milking time. Therefore, it makes sense for farmers to avoid shady farmers and deal with genuine farmers, who provide honest information, keep reliable records, and take good care of animals—animals from such farmers are deemed to have less problems. Hence, should scientists and government officials aim to deliver recommendations on a disease control to farmers, it is necessary to understand farmers' holistic approach to biosecurity.

### Why Do Farmers Change Their Farming Practices?

It was evident that farmers made a substantial behavioral change after one or multiple “trigger events” identified by the Trigger Change Model. These triggering events included three types already discussed by Sutherland et al. (31). While these three types are relatively infrequent events (e.g., devastating disease, succession and new regulations), we point out that the frequency, and even rareness, of events is not necessarily an important characteristic of trigger events. Our study suggested that relatively frequent events can be also a trigger: farm relocation due to the share-milking system specific to New Zealand can also work as a trigger event. We argue that tensions between a land-owner and share-milker, likely due to the difference in their farming subjectivities, play an important role in inducing a behavioral change. Although this system is specific to New Zealand, we postulate a similar tension can occur in any other countries because a farming system often consists of multiple actors including family members, staff, and neighbors. This suggests that routine farming practices may also be considered triggers. Moreover, it points to the importance of understanding different subjectivities within a farm system because a conflict felt by one party (e.g., share-milker) may be different from that of the other party (e.g., land-owner). The immediate implication is that we need to be careful in designing quantitative studies of behavioral change because questionnaire studies often only collect information from one person on the farm. Further studies are warranted to understand how the coexistence of multiple subjectivities within a farm influence the decision making of a whole farm.

Interestingly, it was evident that farmers often demonstrated their frustrations, stress, and emotions associated with triggering events when they were explaining their behavioral changes. Previous studies on stressors on farmers listed a disease outbreak as one of the most stressful events to farmers (53, 55). A Swedish study also reported that a higher disease (mainly mastitis) incidence rate was associated with farm workers being more frequently exposed to psychosocial stressors (56). Introducing a disease or undesirable cows seemed to act as a trigger event because it posed significant stress on farmers—be it a serious workload to deal with the consequence or the loss of freedom of doing what farmers used to do. We therefore postulate the degree of stress and emotional impact that trigger events pose on farmers is an important characteristic which may determine their behavioral consequences. While we cannot conclude this hypothesis based only on this study, there is a wealth of knowledge in the psychology discipline that shows “coping strategies” may be employed to diminish the physical, emotional, and psychological burden that is linked to stressful events (57).

Coping may take different forms depending on various factors including the affected person's perception of the stressful event, perceived capacity to deal with the event, belief, resources such as supporting networks, and the person's situation (57, 58). Psychological studies traditionally categorize these forms into two types: engagement (approach) and disengagement (avoidance) (59). Whereas, engagement coping strategies involve reactions and attentions toward the stressor (stressful events), disengagement strategies involve an attempt to stay away from the stressor. In the context of livestock purchasing behaviors, both forms can, for instance, lead to cessation of livestock purchasing. While some farmers may stop purchasing because they believe they can stay away from introducing a disease (disengagement), others may be more engaged in understanding disease and decide the best solution is to stop purchasing animals (engagement). Although these two strategies may lead to the same behavior, attitudes toward disease control in general may differ between the two. We make it clear that it is not our intention to categorize behavioral changes identified in this study within this coping framework. Rather, we suggest that it is not the outcome of behavioral change that are particularly relevant when understanding a behavioral change—what matters is the process and the context in which a change occurs, as we further discuss below.

### How Do Farmers Change Their Practices?

Our analysis suggested it is challenging to predict whether a minor or major behavioral change occurs after given trigger events: the change seems to be highly context-dependent. Sutherland et al. discussed that farmers are likely to analyse a message or situation differently between when they are in the path-dependent phase and when they just experienced trigger events (31). They argue that peripheral route processing occurs in the path-dependent phase, where farmers assess a message or situation superficially, leading to only an incremental change. On the other hand, after trigger events, farmers use central route processing, where they actively assess available messages and information, leading to a substantial behavioral change. Nevertheless, the real process of a behavioral change seems more complex. For instance, as exemplified by the quote of a farmer who described the experience of dealing with bTB as “fighting fire,” a substantial socio-emotional shock due to trigger events may prohibit farmers from indulging in central route processing. Or, disease outbreak situations such as the current *Mycoplasma bovis* outbreak in New Zealand do not allow farmers to adopt different farm practices due to an imposition of new legislation. Therefore, in general, an incremental change may occur in response to trigger events and a major change may occur without these events. Together, this suggests that it is not outcomes that are particularly relevant when understanding a behavioral change—what really matters is the process and the context in which a change occurs. Our suggestion is therefore to tie the characteristics of events and the characteristics of situations in which these events occur such as cowshed culture, farm financial status, farmers emotion toward the events [e.g., fatalistic against disease, see (60, 61)], and how much support farmers received for the event [e.g., whether farmers have an access to specific instructions, (62)].

### How Does Individual Farmer's Trading Influence an Overall Movement Network Structure?

As we have already seen, stock agents play an essential role among New Zealand dairy farmers. Here, we discuss how such a system also significantly contributes to generating a larger-scale livestock movement network, using a livestock movement in relation to bTB risk as an example. We have previously reported that the frequency of livestock movement from bTB high risk to bTB low risk regions is much lower than expected (10). Our interpretation was that New Zealand dairy farmers may avoid purchasing animals from bTB high risk regions (e.g., West Coast). Nevertheless, the stock agent system provides an alternative explanation.

This trading system results in the majority of New Zealand farmers not having an option to purchase from high-risk regions for several reasons. First, livestock trading in these regions is not extremely profitable for stock agents. This is because stock agents earn money proportional to the total price that buying farmers pay to the seller, but West Coast farmers usually only have a small number of surplus animals to sell because of its severe and wet climate. Second, stock agents who are looking for a large number of animals are unlikely to try to purchase animals from West Coast: it is logistically easier for agents to secure a required number of animals from a single farm rather than gathering a small number of animals each from multiple herds. These factors together limit the number of animals sold from this region to other regions, which in turn leads to animals being traded within the bTB high risk region. The apparent risk-averse livestock movement pattern therefore does not necessarily mean that farmers are intentionally avoiding risky trading. This emphasizes that there are complex factors and actors that are involved in shaping an observed livestock movement network.

We speculate that movement network structure remains similar if farmers keep using the same agent and the supply and demand of livestock does not change dramatically: this is because, again, stock agents often match sellers and buyers locally. A significant change in network structures, however, can occur if, for instance, farms that sell a large number of animals change their stock agents and/or agent companies; this will generate new trade partners, changing a whole network structure. Therefore, although our study focused on farm-level change in trading practice, it is also important to understand how livestock movement patterns change collectively as a system in response to trigger events such as the current *Mycoplasma bovis* outbreak in New Zealand.

## Conclusion

Farmers' livestock purchasing practices appear to be deeply embedded in cowshed culture, which is shaped by physical infrastructure, natural environment, and interactions between animals and farmers. As a result, traditional behavioralist approaches that link farmers' attitudes toward biosecurity and their behaviors may dismiss important aspects of farmers decision making on livestock trading. Drawing on the Trigger Change Model, we showed how trigger events disrupt farmers' established purchasing practices. In response to shock imposed by triggers, farmers reorganize their practices and may develop a more holistic purchasing strategy to reduce a disease introduction risk. However, the impact of triggers seems to be largely context-dependent. Using voluntary schemes such as providing disease status of source farms has attracted great interest as a driver of behavioral change. One hopes such schemes may be easily integrated into an existing farm practice, however, we speculate such an integration is challenging for many farmers due to path-dependency. These schemes may therefore fail to deliver their intended behavioral changes without a greater understanding on trigger events: do these schemes act as a trigger? How do different triggers work in different situations? How do farmers seek support to overcome socio-emotional and economic impacts posed by triggers? How does this support influence on behavioral change amongst farmers? Answering these questions requires a paradigm shift in how epidemiologists frame farming behaviors—they are much more than a biosecurity question.

## Data Availability Statement

The datasets for this manuscript are not publicly available because of the privacy nature of the interview data. Requests to access the datasets should be directed to AH (A.Hidano@massey.ac.nz).

## Ethics Statement

The studies involving human participants were reviewed and approved by peer review at Massey University under Ethics Notification Number 4000016617 and judged to be low risk. Consequently it has not been reviewed by one of the University's Human Ethics Committees. The researchers named in this document are responsible for the ethical conduct of this research. If you have any concerns about the conduct of this research that you want to raise with someone other than the researchers, please contact Dr. Brian Finch, Director (Research Ethics), email: humanethics@massey.ac.nz. The patients/participants provided their written informed consent to participate in this study.

## Author Contributions

AH, MG, and GE conceived and designed the study, wrote the paper with significant intellectual input from GE, and provided approval for publication of the content. AH conducted a field interview. AH and MG secured funding for the study. AH and GE analyzed and interpreted data.

### Conflict of Interest

The authors declare that the research was conducted in the absence of any commercial or financial relationships that could be construed as a potential conflict of interest.
